# The disappearance of gastric metastasis and liver metastasis in non-small cell lung adenocarcinoma is due to osimertinib

**DOI:** 10.1007/s00432-023-05386-7

**Published:** 2023-09-11

**Authors:** Yun Wang, Chao Yan, Chuantao Zhang, Enhao Yu, Kai Wang, Xiangyong Liu, Jie Yu, Chunyang Zhou, Aijie Yang

**Affiliations:** 1 Department of Radiotherapy, Qilu Hospital (Qingdao), Cheeloo College of Medicine, Shandong University, 758 Hefei Road, Qingdao, 266035 Shandong China; 2 The Affiliated Hospital of Qingdao University, Qingdao University, Qingdao, 266003 China

**Keywords:** Non-small cell lung adenocarcinoma, Gastric metastasis, Oxitinib

## Abstract

**Purpose:**

Gastric metastasis of lung cancer is rare, and the cases of disappearance of gastric metastasis and liver metastasis caused by oxitinib treatment have not been reported.

**Methods:**

A 47-year-old male patient with no history of diabetes, hypertension or smoking presented with chest discomfort after eating. At the time of consultation, the diagnosis of adenocarcinoma of the right lower lobe of the lung with liver and gastric metastasis was considered by pathological examination of biopsy of the fundus of the stomach near the cardia, pathological examination of CT-guided lung aspiration and pathological examination of liver occupancy aspiration, combined with immunohistochemical results. He was found to have exon 19 deletion in next generation sequencing. We performed osimertinib on him (EGFR–TKI) systemic therapy, followed by local radiation therapy to the right lower lung primary lesion.

**Results:**

After systemic treatment with osimertinib and local radiotherapy of the primary site, the metastases disappeared and the primary site showed post-radiotherapy changes, and the evaluated efficacy was complete remission.

**Conclusions:**

This is the first report to our knowledge of a patient who presented with gastric and hepatic metastases from lung cancer and achieved complete remission with osimertinib and local radiotherapy, with good quality of life, which also provides a basis for future clinical work and is of great significance.

## Introduction

The diagnosis and treatment of advanced non-small cell lung cancer (NSCLC) have changed dramatically since the discovery of several oncogenic driver mutations [e.g., epidermal growth factor receptor (EGFR) and mesenchymal lymphoma kinase] and the advancement of research into corresponding targeted therapies (Benjamin et al. 2022). The first breakthrough in identifying a population of lung cancer patients likely to respond to molecularly targeted therapy was made in 2004 with the identification of EGFR activating mutations, and since then the field of individualized lung cancer drug use has grown rapidly. EGFR mutations occur in approximately 40–60% of Asian patients and nearly 10–20% of patients with white NSCLC (Hsu et al. 2018). Epidermal growth factor receptor tyrosine kinase inhibitors (EGFR–TKI) have been approved for the treatment of advanced non-small cell lung cancer (NSCLC) carrying EGFR-activating mutations. Osimertinib is a third-generation, irreversible, oral epidermal growth factor receptor (EGFR) tyrosine kinase inhibitor that was evaluated in the FLAURA trial compared to standard EGFR–TKI, gefitinib or erlotinib, leading to FDA approval of osimertinib in April 2018 as a first-line treatment for NSCLC (Tatineni et al. 2023).

Gastric metastases from lung cancer are relatively rare. In fact, its frequency range is from 0.19 to 5.1%. The most common sites of extrapulmonary dissemination include the liver (35%), bone (25%), adrenal glands (22%), kidney (10–15%) and pericardium (20%) (Ozdilekcan et al. 2010). However, a relatively high incidence of postmortem lung adenocarcinoma gastric metastases has been reported, ranging from 4.7 to 14% (Antler et al. 1982). Therefore, this report describes a case of lung adenocarcinoma with gastric metastasis and liver metastasis treated with osimertinib after the disappearance of gastric metastases and liver metastases.

## Case

**Fig. 1:**
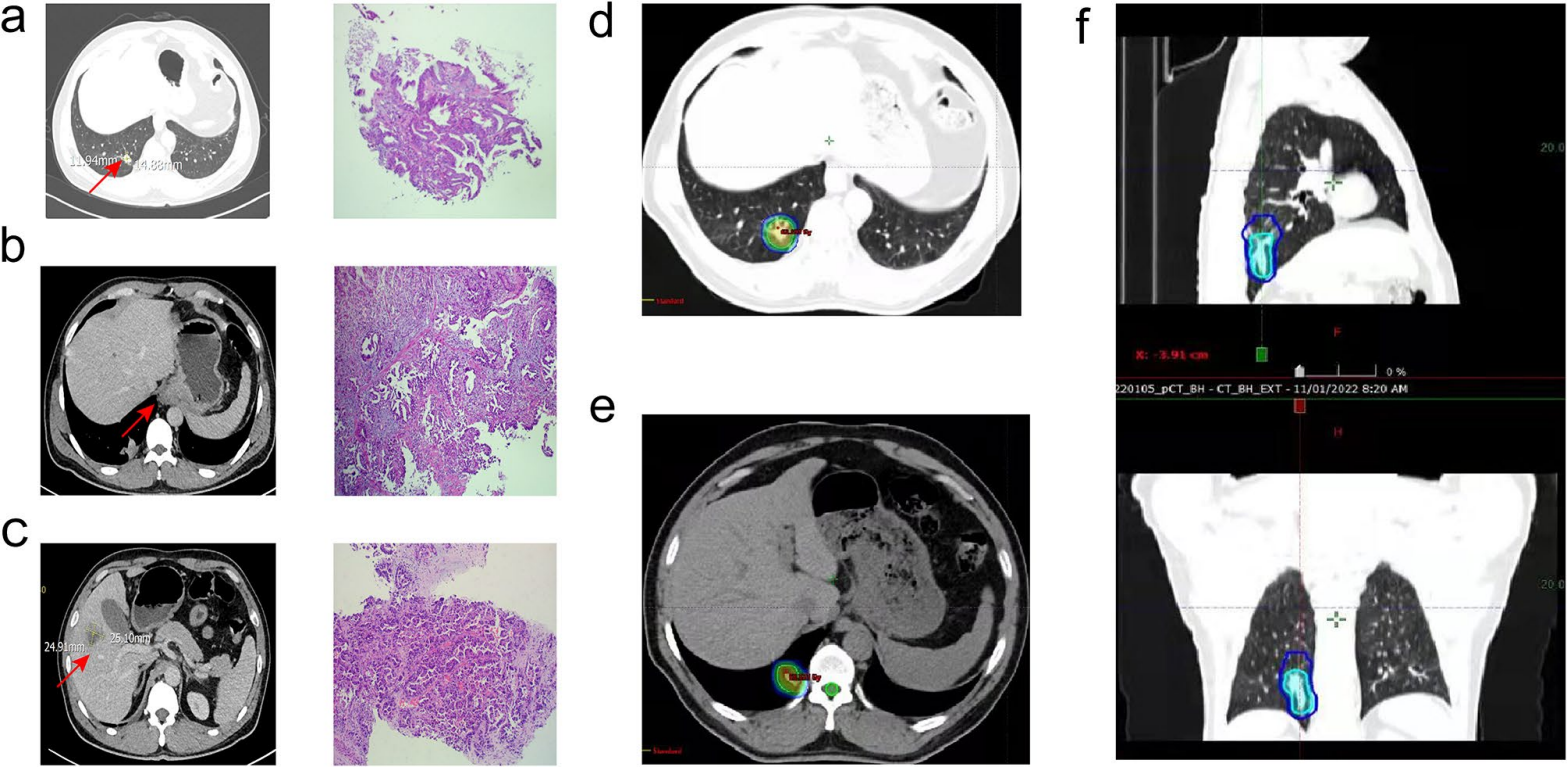
**a** Histopathological examination of lung puncture on August 17, 2023; **b** histopathological examination of gastric puncture on August 17, 2023; **c** histopathological examination of liver puncture on August 20, 2023; **d**–**f** primary focus of the right lower lung was treated with DIBH rotating intensity modulated radiotherapy technology, on January 6, 2022

**Fig. 2:**
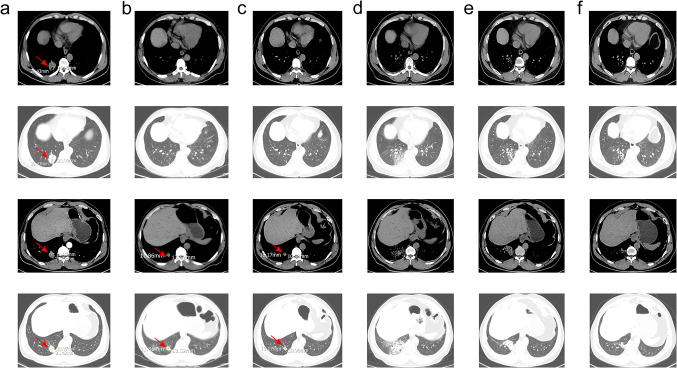
**a** Screenshot of the largest diameter of lung lesions in August 2021; **b** screenshot of the largest diameter of lung lesions in October 2021; **c** screenshot of the largest diameter of lung lesions in January 2022; **d** screenshot of the largest diameter of lung lesions in March 2022; **e** screenshot of the largest diameter of lung lesions in June 2022; **f** screenshot of the largest diameter of lung lesions in March 2023

**Fig. 3:**
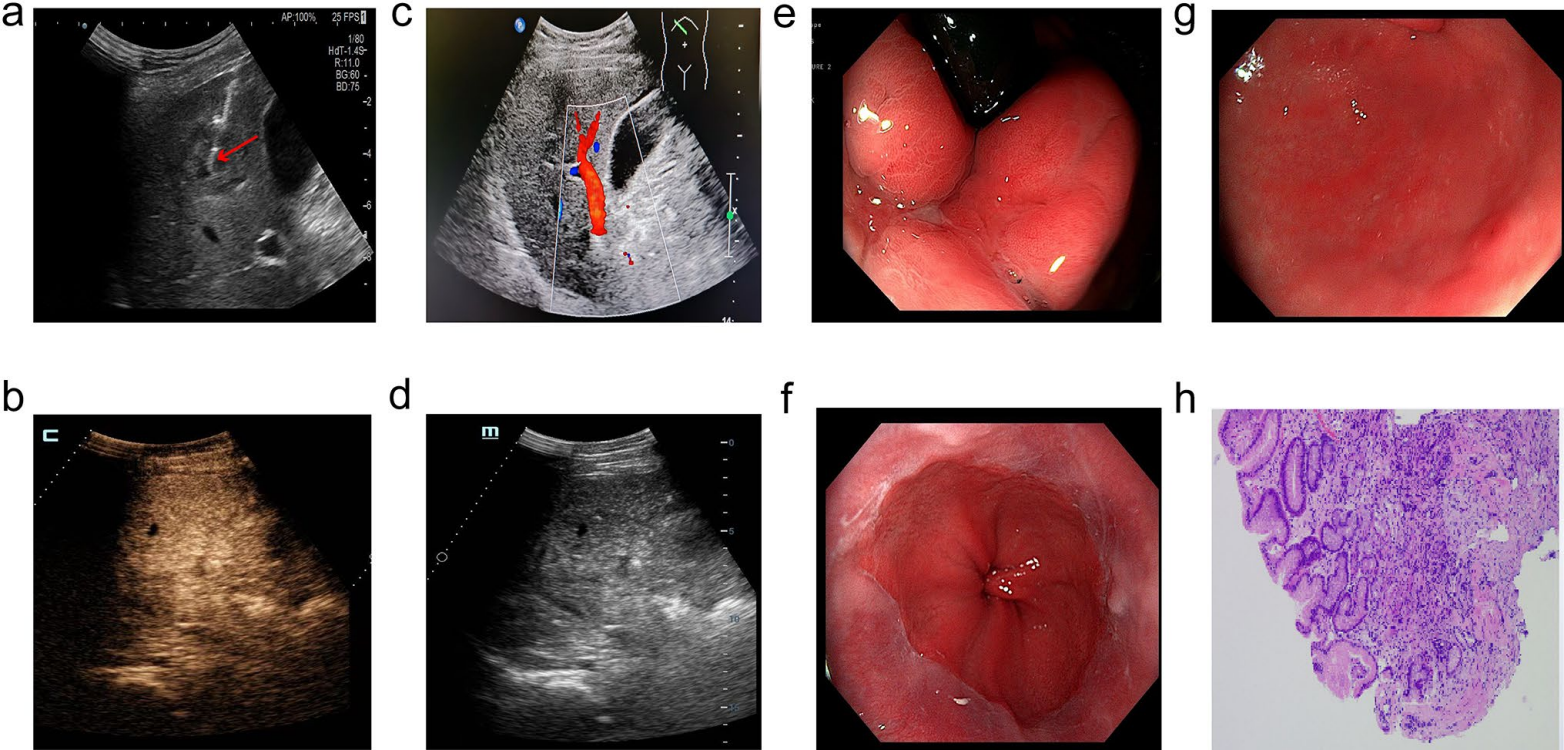
**a** Ultrasound-guided liver space-occupying puncture, on August 20, 2021; **b** liver ultrasound was re-examined in March 2023; **c**, **d** June 2022 contrast-enhanced ultrasound examination of liver; **e**–**g** electronic gastroscope was performed on November 28th, 2022, and **e**, **f**, **g** were cardia, esophagus and antrum under electronic gastroscope respectively; **h** histopathology of biopsy near the fundus of stomach and cardia

A 47-year-old Chinese male patient with no history of smoking (passive smoking only, further questioning of personal history revealed that the patient's father smoked during his 0–13 years and then quilt smoking for physical reasons.) was admitted to the hospital with apparent cause of chest discomfort after eating and feeling of obstruction to eating. Computed tomography (CT) scans of the chest, abdomen and pelvis were performed after admission, showing a lesion in the lower lobe of the right lung with a high likelihood of lung cancer, an abnormally enhancing foci in the parietal lobe of the liver with metastases not excluded, and a hypodense foci in the lower segment of the right anterior lobe of the liver (Fig. 1a–c). Electrogastroscopy showed chronic atrophic gastritis with erosion and a large mass in the fundus of the stomach near the cardia with ulcerated, brittle surface that bleeds easily when touched. Lung CT-guided puncture biopsy, immunohistochemistry ALK (D5F3) (−), CD56 (−), CDX2 (−), CK20 (−), CK5/6 (−), CK7 (+), EGFR (+), Ki67 (hot zone + 40%), P40 (−), TTF-1 (+), Villin (−), combined with immunohistochemistry, led to the diagnosis of adenocarcinoma of the lung (Fig. 1a). Gastroscopic biopsy pathology showed: adenocarcinoma in the fundus near the cardia, immunohistochemistry CDX2(−), CK20(−), CK7(+), NapsinA(+), TTF-1(+), Villin(−), and lung adenocarcinoma metastasis was considered in combination with medical history and immunohistochemistry (Fig. 1b). Pathological examination of liver puncture biopsy with immunohistochemistry TTF-1(+), NapsinA(+), CK7(+), CDX-2(−), villin(−), CK20(−), HepPar-1(−), CK19(foci +), Ki-67(+, 40%), CD10(−), combined with medical history and immunohistochemistry, supported liver metastasis from lung adenocarcinoma (Figs. 1c, 3a). Clinical tumor staging was evaluated as cT2N2M1 stage IV, Mediastinal lymph node metastasis, cardia metastasis, and liver metastasis. Because of the patient and his family urgently demanded early treatment, he underwent 1 cycle of chemotherapy pemetrexed 1.0 g d1 + carboplatin 0.5 g d1 chemotherapy On August 26, 2021. After one cycle of treatment, the patient developed cough and yellow phlegm. Laboratory tests showed that the patient had third-degree bone-marrow suppression, and CRP and PCT were all increased. Considering the infection after bone marrow suppression, the possibility of bacterial infection was high. According to the experience, it was improved after anti-infection treatment with moxifloxacin and rabobarbital. On August 28th, 2021, gene test results back showed: TMB:10.00Muts/Mb (TMB-L), microsatellite stable MSS, EGFR NM-005228.4 exon19 c.2235-2249del, TP53 mutation. Patients subsequently started osimertinib 80 mg qd targeted therapy from October 2021. Months of efficacy were evaluated as PR based on computed tomography (CT) scans of the chest, abdomen and pelvis before targeted therapy and computed tomography (CT) scans of the chest, abdomen and pelvis after targeted therapy in patients (Fig. 2a–f). In order to delay the development of tumor resistance, radiation therapy was performed for the primary foci of the right lower lung from January 6, 2022 (Fig. 1d–f), applying the DIBH rotational intensity-modulated radiotherapy technique, and a total of PTV1 DT 60GY/3GY/20F and PTV2 DT 50GY/2.5GY/20F were given, and the process was smooth. The lung CT was regularly reviewed after radiotherapy, and the lesion was significantly reduced compared with the previous one, and the efficacy was evaluated as PR (partial response) (Fig. 2d–f). In June 2022, radiofrequency ablation of intrahepatic metastatic lesions was proposed, and the intrahepatic lesions were found to disappear, so radiofrequency ablation was not performed (Fig. 3b–d). Continue to take oral osimertinib, 2022-11-28 gastroscopy showed: cardia: 40 cm from the incisor, see a size of about 0.2 × 0.3 cm ulcer-like lesion, the bottom covered with white moss, electronic staining under the observation of local mucosal surface structure irregular, biopsy 1 piece. Pathology showed (cardia) chronic atrophic gastritis with inflammatory infiltrate (++), focal activity (++), atrophy (+), no intestinalization, focal mucosal granulation tissue formation, surface covered with inflammatory exudative necrosis, consistent with ulcer floor changes, HP (special stain-) (Fig. 3e–h). The efficacy was evaluated as CR (complete response) based on gastroscopy and pathology report, and the latest enhanced CT of the chest and upper abdomen, liver ultrasound (Figs. 2f, 3c). The patient is still taking oral osimertinib 80 mg daily and has regular review of whole body CT, and still no progression. We will also continue to follow the changes in this patient's condition.

## Discussion

Gastric metastasis from lung cancer is a relatively rare clinical phenomenon. It was found that gastric metastasis occurred in 0.7% of 1180 autopsies of cancer patients, and about 17 cases presented with gastric metastatic conditions (Menuck and Amberg 1975). Breast cancer, lung cancer and melanoma were the most common primary tumors, while gastric metastasis from lung cancer was much rarer, with squamous cell carcinoma (SCC) cells being the most common type of gastric metastasis (Menuck and Amberg 1975). The detection of gastric metastases from lung cancer depends on the patient's clinical presentation and the evaluation method used. The most common clinical manifestations of the gastrointestinal tract are abdominal discomfort, abdominal pain, bloating, and bleeding, and the value of CT for depicting the structural anatomy is widely understood and applied in the clinic to identify abnormalities in a noninvasive manner (Pezzuto et al. 2013). In the case described in this study, the reason why the relatively rare case of gastric metastasis from lung cancer could be considered depended on the symptoms of epigastric discomfort related to eating and drinking presented by the patient and the findings of the CT scan (Pezzuto et al. 2013). Gastric metastasis, although rare, must be considered when the clinical presentation of the lung cancer patient and the results of the evaluation methods support this rare metastasis (Pezzuto et al. 2013_)._ Based on a review of the literature, it is considered that although the mechanism of gastric metastasis from lung cancer has not been clearly elucidated, the possibility of hematogenous dissemination through the rich vasculature of the stomach is high considering the anatomical structure of the stomach, but this conjecture has also not been clearly confirmed (Maeda, et al. 1992). In addition, considering that after lung cancer metastasis to the stomach, stomach discomfort symptoms may occur, and these symptoms may be aggravated by chemotherapy, thus affecting the patient's subsequent treatment, while the milder gastrointestinal response of osimertinib than chemotherapy is more conducive to improving the patient's motivation for treatment. The prognosis of gastric metastases from lung cancer is reported to be very poor, with a mean survival time of 96.5 days after initial diagnosis (Kim et al. 2009). In the present case, no further cancer cells were detected after systemic treatment with osimertinib and a repeat gastroscopy with pathological biopsy, which also reaffirmed the clinical utility value of this case report.

Osimertinib is a third-generation TKI. The FDA approved osimertinib as first-line treatment for NSCLC carrying EGFR exon 19 deletion or exon 21 L858R mutation in April 2018 after comparing the efficacy of osimertinib with standard treatment through the FLAURA trial (Tatineni et al. 2023; Kim et al. 2009). Reports in the literature show that osimertinib, a third-generation, CNS-permeable, oral EGFR TKI, has shown good efficacy in patients with brain metastases regardless of T790 M (Lee et al. 2020). This article is also the first to report the disappearance of metastases after the use of osimertinib for gastric metastases and liver metastases from pulmonary malignancies.

The results of the study demonstrated a significant improvement in progression-free survival (PFS) when patients with EGFR-mutated advanced NSCLC were treated with an EGFR tyrosine kinase inhibitor (TKI) monotherapy compared to standard chemotherapy (Pezzuto et al. 2017). Thus, the detection of genetic mutations associated with lung cancer and the use of less toxic, better tolerated and more effective biologic drugs that match the target are improving the prognosis of lung cancer patients (Pezzuto et al. 2017). The deletion of exon 19 and the L858R mutation in exon 21 are the EGFR alterations that show the best sensitivity to EGFRTKI therapy. Eastern NSCLC patients responded better to this treatment compared to the West (Pezzuto et al. 2017). Tyrosine kinase inhibitors (TKI) have been reported to significantly improve progression-free survival (PFS) in metastatic non-small cell lung cancer (NSCLC) with epidermal growth factor receptor (EGFR) gene mutations compared with systemic therapy alone. However, most eventually develop resistance with a median PFS of 8–12 months, mainly due to acquired resistance in the structural domain of EGFR kinase (Tibdewal et al. 2021; Vokes et al. 2022). Failure mode studies have shown that patients with disease recurrence in the presence of drug-resistant tumor cells at the original site of disease and combined with P53 mutations are more susceptible to EGFR–TKI resistance (Tibdewal et al. 2021; Vokes et al. 2022).

Our review of the literature found that systemic treatment followed by local treatment was beneficial in delaying drug resistance in patients (Al-Halabi et al. 2015). In the present case, local radiotherapy was administered to the patient’s primary lesion in order to delay drug resistance, and the patient was reviewed regularly; as of today, no recurrence has been observed.

This is the first report to our knowledge of a patient who presented with gastric and hepatic metastases from lung cancer and achieved complete remission with osimertinib and local radiotherapy, with good quality of life, which also provides a basis for future clinical work and is of great significance. In conclusion, for patients with stage IV lung cancer with oligometastases, the addition of local therapy after systemic therapy may help delay drug resistance and facilitate survival.

## Data Availability

All data generated or analysed during this study are included in this published article and its supplementary information files.

## References

[CR1] Al-Halabi H et al (2015) Pattern of failure analysis in metastatic EGFR-mutant lung cancer treated with tyrosine kinase inhibitors to identify candidates for consolidation stereotactic body radiation therapy. J Thorac Oncol 10(11):1601–1607 26313684 10.1097/JTO.0000000000000648

[CR2] Antler AS, Ough Y, Pitchumoni CS, Davidian M, Thelmo W (1982) Gastrointestinal metastases from malignant tumors of the lung. Cancer 49:170–172 6274500 10.1002/1097-0142(19820101)49:1<170::aid-cncr2820490134>3.0.co;2-a

[CR3] Benjamin DJ, Haslam A, Gill J et al (2022) Targeted therapy in lung cancer: are we closing the gap in years of life lost? Cancer Med. 10.1002/cam4.4703 35315222 10.1002/cam4.4703 PMC9487872

[CR4] Hsu WH, Yang JCH, Mok TS et al (2018) Overview of current systemic management of EGFR-mutant NSCLC. Ann Oncol 29:i3–i9 29462253 10.1093/annonc/mdx702

[CR5] Kim MS, Kook EH, Ahn SH et al (2009) Gastrointestinal metastasis of lung cancer with special emphasis on a long-term survivor after operation. J Cancer Res Clin Oncol 135:297–301 18512073 10.1007/s00432-008-0424-0 PMC12160283

[CR6] Lee J, Choi Y, Han J, Park S, Jung HA, Su JM et al (2020) Osimertinib improves overall survival in patients with EGFR-mutated NSCLC with leptomeningeal metastases regardless of T790M mutational status. J Thorac Oncol 15:1758–1766 32652216 10.1016/j.jtho.2020.06.018

[CR7] Maeda J, Miyake M, Tokita K et al (1992) Small cell lung cancer with extensive cutaneous and gastric metastases. Intern Med 31:1325–1328 1338292 10.2169/internalmedicine.31.1325

[CR8] Menuck LS, Amberg JR (1975) Metastatic disease involing the stomach. Am J Dig Dis 20:903–913 1190198 10.1007/BF01070875

[CR9] Ozdilekcan C, Songür N, Memis L, Bozdogan N, Koksal AS, Ok U (2010) Lung cancer associated with a single simultaneous solitary metastatic lesion in stomach: a case report with the review of literature. Tuberk Toraks 58:78–84 20517733

[CR10] Pezzuto A, Mariotta S, Fioretti F, Uccini S (2013) Metastasis to the colon from lung cancer presenting with severe hyponatremia and dyspnea in a young male: a case report and review of the literature. Oncol Lett 5(5):1477–1480 23761813 10.3892/ol.2013.1208 PMC3678879

[CR11] Pezzuto A, Terzo F, Graziani ML, Ricci A, Bruno P, Mariotta S (2017) Lung cancer requires multidisciplinary treatment to improve patient survival. Oncol Lett 14(3):3035–3038 28928841 10.3892/ol.2017.6511 PMC5588536

[CR12] Tatineni V, O’Shea PJ, Ozair A, Khosla AA et al (2023) First- versus third-generation EGFR tyrosine kinase inhibitors in EGFR-mutated non-small cell lung cancer patients with brain metastases. Cancers (basel) 15(8):2382 37190312 10.3390/cancers15082382 PMC10137202

[CR13] Tibdewal A et al (2021) Protocol for a phase II randomised controlled trial of TKI alone versus TKI and local consolidative radiation therapy in patients with oncogene driver-mutated oligometastatic non-small cell lung cancer. BMJ Open 11(2):e041345 33589450 10.1136/bmjopen-2020-041345 PMC7887350

[CR14] Vokes N et al (2022) Concurrent TP53 mutations facilitate resistance evolution in EGFR-mutant lung adenocarcinoma. J Thorac Oncol 17(6):779–792 35331964 10.1016/j.jtho.2022.02.011 PMC10478031

